# Estimation of Covid-19 lungs damage based on computer tomography images analysis

**DOI:** 10.12688/f1000research.109020.1

**Published:** 2022-03-17

**Authors:** Martin Schätz, Olga Rubešová, Jan Mareš, David Girsa, Alan Spark

**Affiliations:** 1 Department of Computing and Control Engineering, University of Chemistry and Technology, Prague, 166 28, Czech Republic; 2 Department of Radiodiagnostics 3FM CU and UHKV, Charles University 3rd Faculty of Medicine, Prague, 100 34, Czech Republic

**Keywords:** Computed Tomography, Image Analysis, ImageJ, Covid-19, Lungs

## Abstract

Modern treatment is based on reproducible quantitative analysis of available data. The Covid-19 pandemic did accelerate development and research in several multidisciplinary areas. One of them is the use of software tools for faster and reproducible patient data evaluation. A CT scan can be invaluable for a search of details, but it is not always easy to see the big picture in 3D data. Even in the visual analysis of CT slice by slice can inter and intra variability makes a big difference. We present an ImageJ tool developed together with the radiology center of Faculty hospital Královské Vinohrady for CT evaluation of patients with COVID-19. The tool was developed to help estimate the percentage of lungs affected by the infection. The patients can be divided into five groups based on percentage score and proper treatment can be applied

## Introduction

The covid pandemic that has affected in recent months has revealed a number of strengths and weaknesses in health systems around the world.

One of the key ideas is a quick and accurate diagnosis of the patient, which was problematic in congested hospitals. Software engineering and image processing methods could be helpful in speeding up and refining patient diagnosis. Especially in radiological and radiodiagnostic workplaces, where a large part of diagnostic processes take place over image data (CT, NMR, X-ray). Various software tools have been used for this purpose for years. In general, it is possible to divide them into two groups:

• universal software packages: used for general analysis of image data such as filtering, smoothing or image registration
• software tools “made to measure”: very specific software tools for analysis of rare diseases

The first group of tools is represented mostly by software integrated into packages supplied by the tomograph developer. It is possible to mention a software tool for CT image preprocessing and automated analysis of three standard phantoms ^1^ or software tool for metal artifacts reduction in dental care. ^2^

The second group of tools is from both the research and application point of view much more interesting. It is necessary to state that only a small part of them is applied in a real clinical environment. It is possible to mention a tool for analysis of GPA disease using image registration and self-organizing maps, ^3^ or a tool for analysis of peripheral bypass grafts. ^4^ Many research groups focused on precise measurement of pathological findings, 3D analysis, or volumetric analysis. ^5^^,^^6^

Moreover, some papers deal with image fusions from different scanners e.g. combination of data from CT, PET/CT, SPECT/CT, or MR. ^7^^,^^8^ Thus, the topic of CT image analysis of “covid lungs” is important from both the research point of view (there is still room for further research in precise semi-automatic analysis) and the clinical point of view. Therefore, the aim of this study is to develop a semi-automatic software for “covid lungs” CT image analysis, based on knowledge presented in Ref. 9. The authors present the idea based on the correlation between the degree of lung involvement and the course of the disease. The global score (0–25) of lung involvement is calculated based on the extent of each lobar involvement score (0: 0%, 1: <5%, 2: 5-25%, 3:26 – 50%, 4:51–75%, 5, > 75%). The authors then introduce the role of CT score for predicting the outcome of SARS-CoV-2 patients. The scoring is highly correlated with laboratory findings, disease severity and mortality. Moreover, it might speed up diagnostic workflow in symptomatic cases.

## Methods

### Image format

The Covid CT estimation tool is based on standard image processing techniques. Our interest is in volume, so the same voxel size is critical for good enough estimation. But it is also important to go through the different types of data we can encounter. In general, the Hounsfield Units (HU) make up the grayscale in medical CT imaging. It is a scale from black to white of 4096 values (12 bit) and ranges from -1024 HU to 3071 HU (zero is also a value). It is defined by the following:

-1024 HU is black and represents air (in the lungs). 0 HU represents water (since we consist mostly out of the water, there is a large peak here). 3071 HU is white and represents the densest tissue in a human body, such as tooth enamel. All other tissues are somewhere within this scale; fat is around -100 HU, muscle around 100 HU, and bone spans from 200 HU (trabecular/spongeous bone) to about 2000 HU (cortical bone).

DICOM files are usually saved in signed 16 bit, with original HU, usually with 3 mm slicing or 0.6 mm slicing CT images. TIFF, however, may have reshaped histogram values to cover the whole range and can preferable be in unsigned 16 bit or 8bit with some loss due to conversion. TIFF values usually lose Z voxel size metadata in conversion (resulting in Z voxel size value of 1), so it is important to reset voxel values. The XY voxel size can be different with each data set, even from the same CT machine. The distribution of intensity values may change with different CT protocols, so some of the processing steps need to be done manually.

### Implementation

The workflow follows the Croney Ethical guidelines for the appropriate use and manipulation of scientific digital images. ^10^

**Figure 1.**
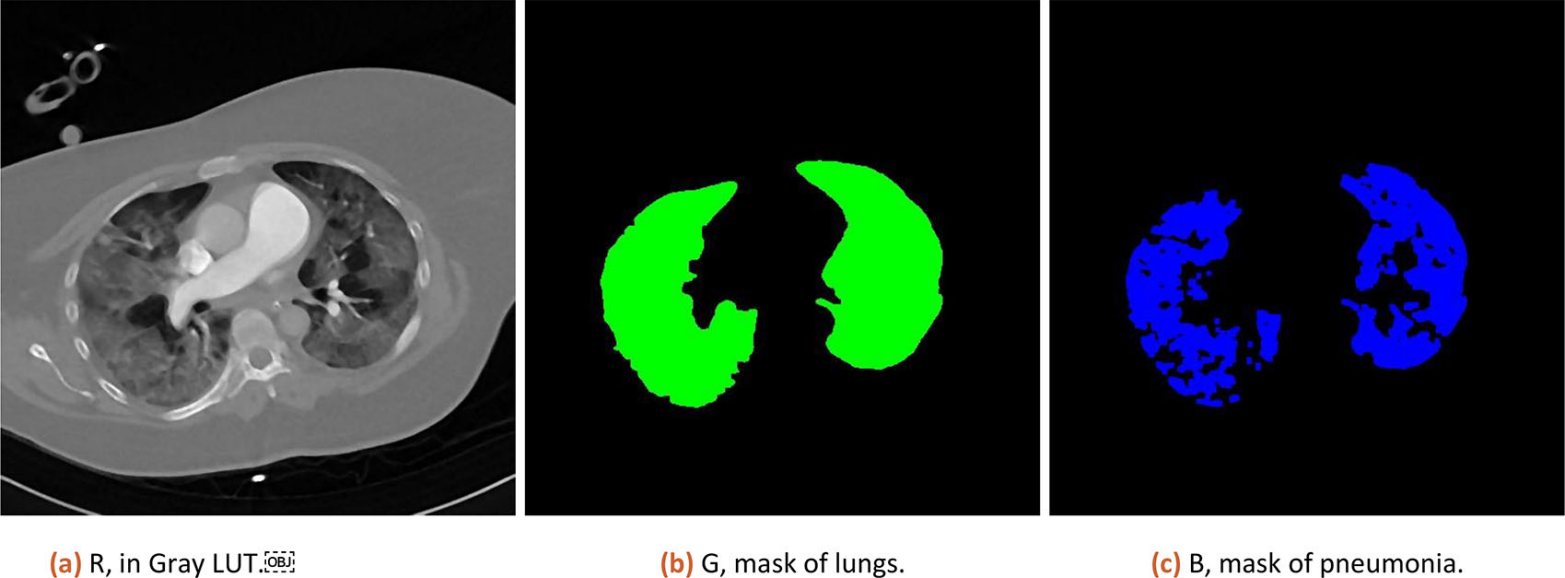
Result of analysis as RGB stack, where Red channel contains CT data, Green channel lung mask and Blue channel pneumonia mask.

The plugin tool is developed in ImageJ macro language, it needs Bio Format plugin to import DICOM files, which comes installed in FIJI. The macro language uses standard image processing techniques and morphological operations to estimate the volume ratio of lungs and pneumonia caused by COVID-19. It allows users to subsequently set up a threshold for pneumonia and lungs, and go through the whole data-set slice by slice and interactively tweak the threshold values. The tool was developed based on demand and with coordination from the Department of Radiology from the Faculty hospital Královské Vinohrady. It is challenging to do any kind of percentage estimate of pneumonia in the lungs just by visually inspecting CT scans stack by stack. Available hardware equipment and local account restrictions had to be taken into account for development tool selection. The ImageJ plugin is a compromise in accuracy and requirements. The workflow is following:

1. DICOM or Tiff stack is imported, the user is prompted to select sub-volume containing only lungs. This step is necessary for the exclusion of other body cavities, which would be otherwise counted as lungs.
2. User manually thresholds background and inside of lungs (air).
3. A lung mask is created by excluding mask components touching the edge of images and by excluding other objects based on size and roundness.
4. User is prompted to select by threshold all areas containing pneumonia (possible parts outside lungs will be excluded).
5. A clean pneumonia mask is created by element-wise multiplication of lung and pneumonia mask.
6. A volume fraction is estimated by voxel count in each mask.
7. Numeric result and coloured visual representation of original data, lung and pneumonia mask is shown to the user as illustrated on Figure 1.
8. A detailed log with tool version, original folder path, score, percentage results, threshold values, and all masks is saved in a folder next to the original image set. Log contains all information needed to do analysis again with same results.

The numeric results in percent is then corrected by subtracting of 3% (median of tissue present in healthy lungs, estimated from 10 patients) and CT is scored based on severity ranged (0:0%; 1, < 5%; 2:5–25%; 3:26–50%; 4:51–75%; 5, > 75%; range 0–5) defined by Ref. 9.

### Operation

**Figure 2.**
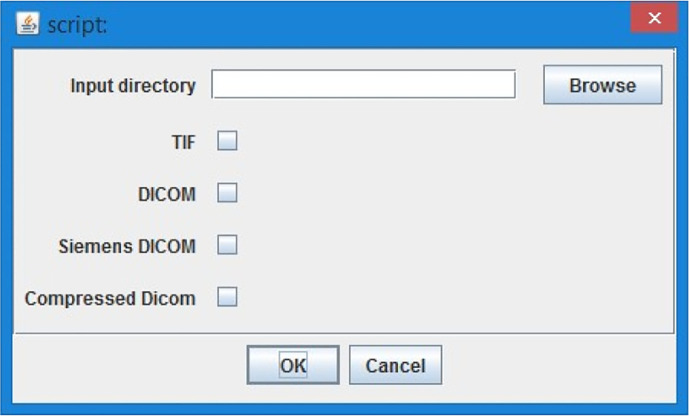
ImageJ Tool, loading data options.

There are several steps during the tool runtime which require user inputs:

1. Select the CT lung data ( Figure 2, TIFF or DICOM file based on the script version) - the CT sequence is opened and user can go through loaded stack in image sequence with a slider or as a video with a play button.
2. “Please find the start of lungs in stack” - user has an option to select the first image with lungs with a slider and confirm the selection with “Ok” button.
3. “Please find the end of lungs in stack” - user has an option to select the last image of lungs selection with the slider and confirm with “Ok” button. The tool works with the images only in between the chosen interval of the lungs stack to minimize the computational effort.
4. “Setup threshold for all but body” - the whole image- exclude the body, shall be highlighted with red colour. The tool makes automatic estimation, and the user can adjust the threshold with the sliders on the histogram. Confirm with the “Ok” button.
5. “Setup threshold of Covid” - the covid threshold shall be highlighted with red colour. The tool makes automatic estimation, and the user can adjust the threshold with the sliders on the histogram. It is not a problem if part of the body (not lungs!) will be chosen together with Covid. The tool automatically subtracts the body threshold from the chosen Covid threshold. Confirm with the “Ok” button.

• After each calculation the tool is adding information to the log window. The log file is automatically saved to the CT data directory. The output lungs and covid masks are saved in TIFF format into an additional folder in the CT data location.
• The tool provides % estimation of Covid damage in the lungs and a semi-quantitative CT score. The score is calculated based on the extent of lobar involvement (0:0%; 1, < 5%; 2:5–25%; 3:26–50%; 4:51–75%; 5, > 75%; range 0–5 based on the medical research “Chest CT score in COVID-19 patients: correlation with the disease severity and short-term prognosis. ^9^

The tool has been tested both 3 mm slicing and 0.6 mm slicing CT images. The results were similar in percentage and the final CT score was the same.

In order to use the tool, the user needs to prepare CT images exported as DICOM or TIFF in preferred view mode and preferably 16-bit representation. The CT images have usually a 12-bit gray-scale representation and an 8-bit conversion would lead to loss of potentially important information or shift of brightness values. The thickness of the CT slice can also contribute to numerical errors in the process, but there was no significant difference in results when processing the same data-set with 3 mm and 0.6 slicing.

The ImageJ software tool available from Zenodo needs an ImageJ installed with Bio-Formats (preferably with version 6.8.0 which we tested) plugin (or FIJI which is a version of ImageJ with an already integrated Bio-Formats plugin).

Minimal requirements for both are Windows XP or later with Java installed, Mac OS X 10.8 or later with Java installed, Ubuntu Linux 12.04 LTS, or later with Java installed. Minimal RAM is based on the size of processed images, in this case, there are multiple images opened at once.

## Use cases

A usability of introduced tools is presented in next sections. A use case for comparison for a CT measured with different slicing setup is presented. Results for a set of 5 CTs evaluated by different users is discussed. Since we were restricted by hardware, two versions of tool were created. One that is working with 8-bit version of images and needs less RAM, and one that works with 16-bit signed images and can load HU units.

### Slice thickness variation

The international standard for saving DICOM files defines 3 mm slicing of CT data as the default way. However resaving data as TIFF (losing voxel information) or using different slice thicknesses (like 0.6 mm slicing) may result in a different result. In theory, 0.6 slicing would provide 5 times more detailed sampling in the Z-axis, however, in practice it is different.

**Figure 3.**
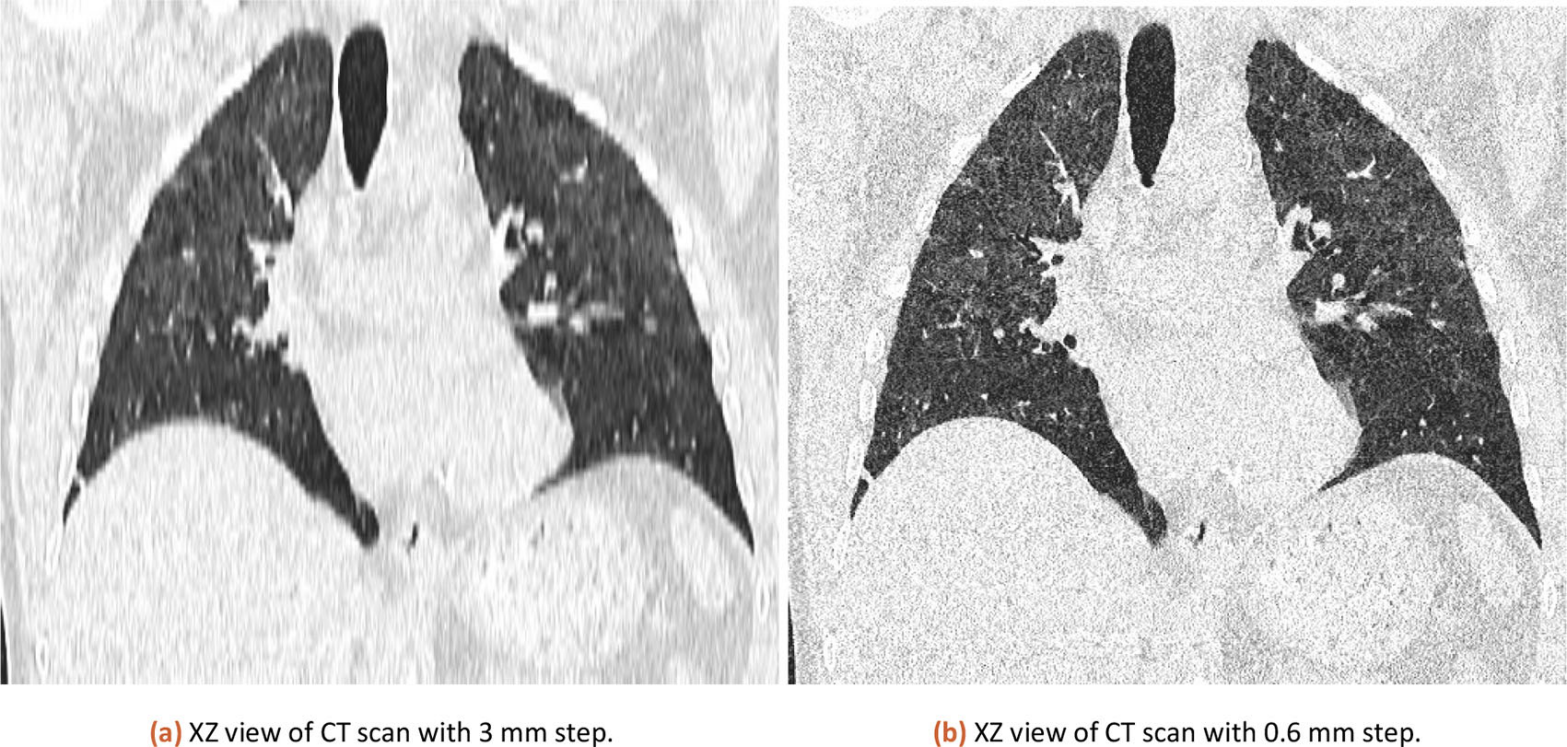
XZ view comparison of 3 mm and 0.6 mm CT.

The same CT dataset exported with 0.6 and 3 mm slices (XZ view for comparison is in Figure 3) was analyzed with our tool with a lung threshold of 0-155 and a pneumonia threshold of 47-115. The results can be found in Table 1. The error from a comparison of 3 mm and 0.6 mm slicing is estimated at 0.58 %. The used CT is available in the attached published dataset as CT1_1 (0.6 mm slicing) and CT1_2 (3 mm slicing).

**Table 1.** Comparison of results from 0.6 mm and 3 mm 8-bit dataset.

Slicing	Lungs slices	Lungs threshold	Pneumoina threshold	Percentage	Scoring
0.6 mm	60-505	0-155	47-115	31.21	3
3 mm	12-101	0-155	47-115	31.79	3

### User inter and intra variability

The biggest challenge in using this tool is an individual perception of images, as each person may see image data fundamentally the same - despite different appearances. Based on this a user can add the biggest bias even though the underlying data analysis is done correctly. The Table 2 contains a comparison of analysis results on 5 different CT datasets provided by the Faculty hospital of Královské Vinohrady. All of the CTs are analysed by users with various experience, the first CT exported with different slicing (also used in Table 1) is analysed by a radiologist (an expert user). The score aims to divide the percentage into groups based on previous research done, ^9^ and should be the deciding factor of future care for patients.

**Table 2.** Independent analysis results.

Dataset	Slicing	Radiologist	Rad. score	User 1	User 1 score	User 2	User 2 score	User 3	User 3 score
CT1_1	0.6 mm	31%	3	50%	3	30%	3	30%	3
CT1_2	3.0 mm	32%	3	55%	4	33%	3	45%	3
CT2	3.0 mm	-	-	10%	2	5%	1	7%	1
CT3	0.6 mm	-	-	41%	3	24%	2	42%	4
CT4	3.0 mm	-	-	64%	4	64%	4	64%	4
CT5	3.0 mm	-	-	2%	1	3%	1	4%	1

## Discussion

The ImageJ/FIJI tool can import various DICOM or TIFF files. Users should be always aware of whenever the saved data are using signed or unsigned bit depth, as unsigned data will shift pixel brightness. The same will happen when exporting data in different bit depth or with a specific CT view. The slicing of the CT dataset also matters, however, the analysis in Table 1 showed that it won’t significantly affect neither the percentage or the score (other CT machines might have different settings). A small case study for user inter and intra variability was made ( Table 2) to evaluate the usability of the proposed tool. Some expected variability in results occur, interesting is inter variability in evaluating CT1 which is 3-5%. The intra variability is more extensive, up to 20%, and points out the fact that users should have at least some training in how to recognize pneumonia in CT images.

## Conclusions

The tool was developed on demand from the Department of Radiology from the Faculty hospital Královské Vinohrady, as it was difficult for them to estimate the percentage and score of pneumonia in the lungs just by visually inspecting CT scans. Available hardware equipment and local account restrictions had to be taken into account for development tool selection. The ImageJ plugin is a compromise in accuracy and requirements. It logs all the user inputs for reproducibility and saves the results of all the steps as TIFF stacks. These masks and images can be used for visual inspection or possibly in the future for more advanced machine learning tools.

This software tool is the first step of a longer journey to create a tool that would be both easy to use for radiologists to diagnose COVID-19 based on CTs and include an advanced image analysis tool for percentage estimation of pneumonia in lungs. The use of open software promises ease of future development, however, it might be beneficial to move from ImageJ to 3D Slicer ^11^ or Napari ^12^ as they offer better tools for 3D visualization and integration of machine learning tools, which we aim to develop and integrate in our future works.

### Limitations

The biggest limitation of this approach is human error, and inter and intra variation of manual selection. The percentage estimation might also be affected by other body cavities filled with air. There might also be a variance in results based on slice thickness, in worst case scenario 20%, but our experiment shows that there is only about 0.58% difference in result between 0.6 and 3mm CT slice thickness. The scoring should also be improved so it is not dependent only on one value (volume percentage), but normalized SHU distribution in the pneumonia area should be also considered.

## Data availability

### Underlying data

Zenodo: CT scans of COVID-19 patients, https://doi.org/10.5281/zenodo.5805939. ^13^

This project contains the following underlying data:

• CT1_1
  – CT1_1_TIFF_06_MM (Single stack 8-bit TIFF data)
• CT1_2
  – CT1_2_TIFF_3_MM (Single stack 8-bit TIFF data)
• CT2
  – CT2_DICOM
  – CT2_TIFF (Single stack 8-bit TIFF data)
• CT3
  – CT3_DICOM
  – CT3_TIFF (Single stack 8-bit TIFF data)
• CT4
  – CT4_DICOM
  – CT4_TIFF (Single stack 8-bit TIFF data)
• CT5
  – CT5_DICOM
  – CT5_TIFF (Single stack 8-bit TIFF data)
• overview.csv (Detailed overview of each CT set.

Data are available under the terms of the Creative Commons Attribution 4.0 International (CC-BY 4.0).

## Software availability

Zenodo: ImageJ tool for percentage estimation of pneumonia in lungs, https://doi.org/10.5281/zenodo.5805989. ^14^

This project contains the following underlying data:

• SEQUENCE_Est_Percentage_CT_16bit_V03_clean.ijm (16bit version)
• SEQUENCE_Est_Percentage_CT_u8bit_V03_clean.ijm (8bit version)
• results.csv (More detailed results of the dataset analysis)

Data are available under the terms of the Creative Commons Attribution 4.0 International license (CC-BY 4.0).
