# Estimation of Covid-19 lungs damage based on computer tomography images analysis

**DOI:** 10.12688/f1000research.109020.3

**Published:** 2025-07-25

**Authors:** Martin Schätz, Olga Rubešová, Jan Mareš, David Girsa, Alan Spark

**Affiliations:** 1 Department of Mathematics, Informatics and Cybernetics, University of Chemistry and Technology, Prague, 166 28, Czech Republic; 2 Department of Radiodiagnostics 3FM CU and UHKV, Charles University 3rd Faculty of Medicine, Prague, 100 34, Czech Republic

**Keywords:** Computed Tomography, Image Analysis, ImageJ, Covid-19, Lungs

## Abstract

Modern treatment is based on reproducible quantitative analysis of available data. The Covid-19 pandemic did accelerate development and research in several multidisciplinary areas. One of them is the use of software tools for faster and reproducible patient data evaluation. A CT scan can be invaluable for a search of details, but it is not always easy to see the big picture in 3D data. Even in the visual analysis of CT slice by slice can inter and intra variability makes a big difference. We present an ImageJ tool developed together with the radiology center of Faculty hospital Královské Vinohrady for CT evaluation of patients with COVID-19. The tool was developed to help estimate the percentage of lungs affected by the infection. The patients can be divided into five groups based on percentage score and proper treatment can be applied.

## Introduction

The covid pandemic that has affected in recent months has revealed a number of strengths and weaknesses in health systems around the world.

One of the key ideas is a quick and accurate diagnosis of the patient, which was problematic in congested hospitals. Software engineering and image processing methods could be helpful in speeding up and refining patient diagnosis, especially in radiological and radiodiagnostic workplaces, where a large part of diagnostic processes take place over image data (CT, NMR, X-ray). Recent advances in image analysis motivate for a more collaborative approach to quantitative analysis since it usually requires expertise in bioimage analysis. ^1^^,^^2^ Various software tools have been used for this purpose for years. In general, it is possible to divide them into two groups:

• universal software packages: used for general analysis of image data such as filtering, smoothing or image registration
• software tools “made to measure”: concrete software tools for analysis of rare diseases

The first group of tools is represented mostly by software integrated into packages supplied by the tomograph developer. It is possible to mention a software tool for CT image preprocessing and automated analysis of three standard phantoms ^3^ or a software tool for reducing metal artifacts in dental care. ^4^

The second group of tools is from both the research and application point of view much more interesting. It is necessary to state that only a small part of them is applied in a real clinical environment. It is possible to mention a tool for analysis of GPA disease using image registration and self-organizing maps, ^5^ or a tool for analysis of peripheral bypass grafts. ^6^ Many research groups focused on precise measurement of pathological findings, 3D analysis, or volumetric analysis. ^7^^,^^8^

Moreover, some papers deal with image fusions from different scanners e.g. combination of data from CT, PET/CT, SPECT/CT, or MR. ^9^^,^^10^ Thus, the topic of CT image analysis of “covid lungs” is essential from both the research point of view (there is still room for further research in precise semi-automatic analysis) and the clinical point of view.

The availability of tools for scientific research remains a challenge for both researchers and end-users. Although access to scientific papers is increasingly open, reproducible resources, code, and data availability is not yet widespread. Access to the results of scientific studies is crucial, but access to the necessary tools makes a real difference. Unfortunately, the code is not often available in open-source form, complete with step-by-step tutorials and opportunities for reporting issues. While software such as ImageJ ^11^ and 3D Slicer ^12^ exists for image analysis, they are geared toward experienced image analysts. They may not be user-friendly for end-users who are not familiar with creating analysis workflows. The end-user often depends on core facilities or available documentation and tutorials for support. The 3D Slicer CT Lungs Analyzer project for lung analysis is still in development and relies on Unet deep learning segmentation, but it is promising. There is a need for a portable, user-friendly software tool for reproducible quantitative analysis of CTs to estimate covid lung pneumonia.

Therefore, the aim of software paper is to present a semi-automatic software for “covid lungs” CT image analysis, based on knowledge presented in Ref. 13. The authors based the idea on the correlation between the degree of lung involvement and the course of the disease. The global score (0–25) of lung score involvement is calculated based on the extent of volume involvement (0: 0%, 1: <5%, 2: 5-25%, 3:26 – 50%, 4:51–75%, 5, > 75%). The authors then introduce the role of CT score in predicting the outcome of SARS-CoV-2 patients. The scoring is highly correlated with laboratory findings, disease severity and mortality. Moreover, it might speed up diagnostic workflow in symptomatic cases.

## Methods

### Image format

The Covid CT estimation tool is based on standard image processing techniques. Our interest is in volume, so the same voxel size is critical for good enough estimation. But it is also important to go through the different types of data we can encounter. In general, the Hounsfield Units (HU) make up the grayscale in medical CT imaging. It is a scale from black to white of 4096 values (12 bit) and ranges from -1024 HU to 3071 HU (zero is also a value). It is defined by the following:

-1024 HU is black and represents air (in the lungs). 0 HU represents water (since we consist mostly out of the water, there is a large peak here). 3071 HU is white and represents the densest tissue in a human body, such as tooth enamel. Materials with higher atomic numbers, such as bones, appear as brighter areas on CT images and are assigned higher HU values (typically between +700 and +3000). All other tissues are somewhere within this scale; fat is around -100 HU, muscle around 100 HU, and bone spans from 200 HU (trabecular/spongeous bone) to about 2000 HU (cortical bone).

DICOM files are usually saved in signed 16 bit, with original HU, usually with 3 mm slicing or 0.6 mm slicing CT images. TIFF, however, may have reshaped histogram values to cover the whole range and can preferably be in unsigned 16 bit or 8bit with some loss due to conversion. TIFF values usually lose Z voxel size metadata in conversion (resulting in Z voxel size value of 1), so it is essential to reset voxel values. The XY voxel size can be different with each data set, even from the same CT machine. The distribution of intensity values may change with different CT protocols, so some of the processing steps need to be done manually.

### Implementation

The workflow follows the Croney Ethical guidelines for the appropriate use and manipulation of scientific digital images. ^14^

The plugin tool is developed in ImageJ macro language. It needs Bio Format plugin to import DICOM files, which comes installed in FIJI. The macro language uses standard image processing techniques and morphological operations to estimate the volume ratio of lungs and pneumonia caused by COVID-19. It allows users to subsequently set up a threshold for pneumonia and lungs, and go through the whole data-set slice by slice and interactively tweak the threshold values. The tool was developed based on demand and with coordination from the Department of Radiology from the Faculty hospital Královské Vinohrady. It is challenging to do any kind of percentage estimate of pneumonia in the lungs just by visually inspecting CT scans stack by stack. The available hardware equipment and local account restrictions had to be taken into account for development tool selection. The ImageJ plugin is a compromise in accuracy and requirements. The scripts are published with the paper. The workflow for 8-bit script version is following:

1) Input and pre-processing
  a) Clear the log and close all open images.
  b) Print the version of ImageJ and the Bio-Formats Macro Extensions being used. If untested version of ImageJ is being used, a warning message is displayed.
  c) Get the user's input for the type of image file to be processed (TIF, DICOM, Siemens DICOM, or Compressed DICOM) and the directory where the images are stored.
  d) Open the selected image files from the input directory.
  e) Get the dimensions of the image stack (width, height, channels, slices, and frames).
  f) Get the user's input on the start and end slices of the lung region.
  g) Duplicate the stack of slices selected for the lung region.
  h) Apply a median filter to the stack of lung slices.
  i) Enhance the contrast of the stack of lung slices. (Only 8 bit version).
  j) Duplicate the stack of lung slices twice, creating two separate stacks for lung thresholding and pneumonia thresholding.
2) Analysis
  a) For the stack of lung slices, convert the image to 8-bit and apply a threshold to remove all but the lung tissue.
  b) Lungs
    i). Get the user's input on the threshold values for the lung tissue.
    ii). Convert the thresholded image to a mask and clean the mask using erode, dilate, and fill holes operation.
    iii). Analyze the selection in the mask to separate the individual lung regions in each stack.
    iv). Save the processed image as a TIF file in a new directory with the date and time as part of the file name.
  c) Pneumonia
    i). Get the user's input on the threshold values for pneumonia.
    ii). Convert the thresholded image to a mask and clean the mask using erode and dilate.
    iii). Combine the mask with the lung mask using an AND operation.
    iv). Analyze the particles in the mask.
    v). Save the processed image as a TIF file in a new directory with the date and time as part of the file name.
3) Evaluation
  a) Create a new image with CT data as channel 1, lung mask as channel 2 and pneumonia mask as channel 3. Save the composite as TIF in the results folder.
  b) Get the total area of lungs and total area of pneumonia for all stacks.
  c) Evaluate percentage of pneumonia area in lungs, and score the results using (0:0%; 1, <5%; 2:5–25%; 3:26–50%; 4:51–75%; 5, >75%; range 0–5) function.
  d) Save log containing information about the whole process in results folder.

**Figure 1.**
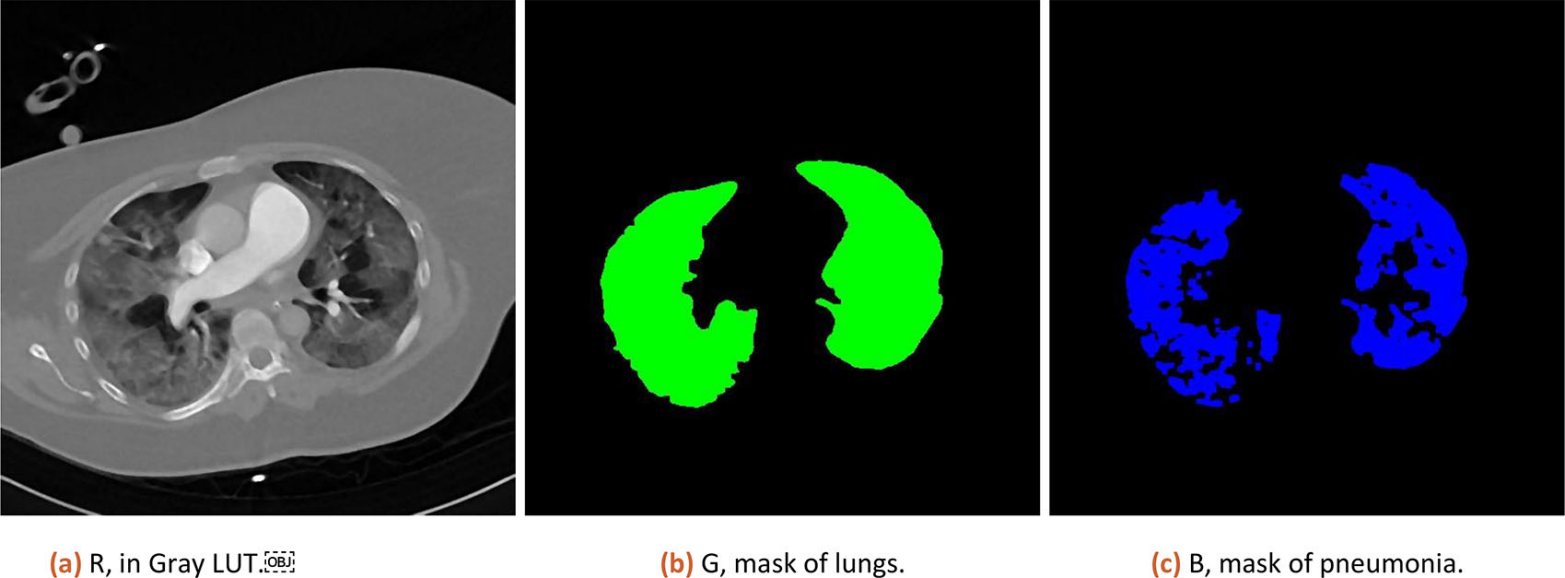
Result of analysis as RGB stack, where Red channel contains CT data, Green channel lung mask and Blue channel pneumonia mask.

Numeric result and composition image representation from step 3.a (original data, lung and pneumonia mask) is shown to the user (as illustrated in Figure 1).

Numeric results in percent are corrected by subtracting 3% (median of tissue present in healthy lungs, estimated from 10 patients) and CT is scored based on severity ranged (0:0%; 1, <5%; 2:5–25%; 3:26–50%; 4:51–75%; 5, >75%; range 0–5) defined by Ref. 13.

### Operation

**Figure 2.**
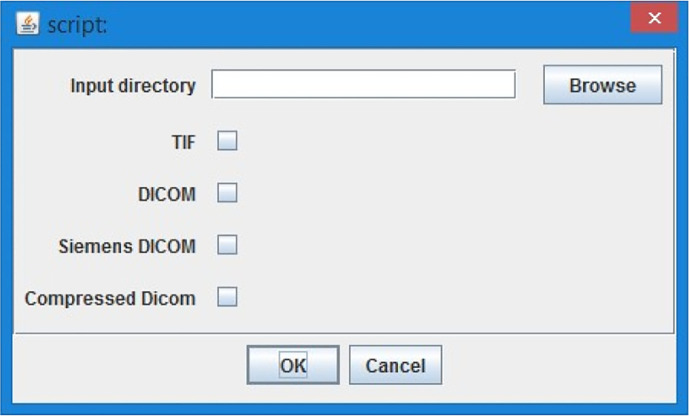
ImageJ Tool, loading data options.

There are several steps during the tool runtime which require user inputs:

1. Select the CT lung data ( Figure 2, TIFF or DICOM file based on script version) - CT sequence is opened and user can go through loaded stack in image sequence with a slider or as a video with a play button.
2. “Please find the start of lungs in stack” - user has an option to select the first image with lungs with a slider and confirm the selection with “Ok” button.
3. “Please find the end of lungs in stack” - user has an option to select the last image of lungs selection with the slider and confirm with “Ok” button. The tool works with the images only in between the chosen interval of the lungs stack to minimize the computational effort.
4. “Setup threshold for all but body” - the whole image- exclude the body, shall be highlighted with red colour. The tool makes automatic estimation, and the user can adjust the threshold with the sliders on the histogram. Confirm with the “Ok” button.
5. “Setup threshold of Covid” - the covid threshold shall be highlighted with red colour. The tool makes automatic estimation, and the user can adjust the threshold with the sliders on the histogram. It is not a problem if part of the body (not lungs!) will be chosen together with Covid. The tool automatically subtracts the body threshold from the chosen Covid threshold. Confirm with the “Ok” button.

• After each calculation the tool adds information to the log window. The log file is automatically saved to the CT data directory. The output lungs and covid masks are saved in TIFF format into an additional folder in the CT data location.
• The tool provides % estimation of Covid damage in the lungs and a semi-quantitative CT score. The score is calculated based on the extent of lobar involvement (0:0%; 1, < 5%; 2:5–25%; 3:26–50%; 4:51–75%; 5, > 75%; range 0–5 based on the medical research “Chest CT score in COVID-19 patients: correlation with the disease severity and short-term prognosis. ^13^

The tool has been tested both 3 mm slicing and 0.6 mm slicing CT images. The results were similar in percentage and the final CT score was the same.

In order to use the tool, the user needs to prepare CT images exported as DICOM or TIFF in the preferred view mode and preferably 16-bit representation. The CT images usually have a 12-bit gray-scale representation and an 8-bit conversion would lead to loss of potentially important information or shift of brightness values. The thickness of the CT slice can also contribute to numerical errors in the process, but there was no significant difference in results when processing the same data-set with 3 mm and 0.6 slicing.

The ImageJ software tool available from Zenodo or GitHub needs an ImageJ (ideally version 1.52v99 or newer) installed with Bio-Formats (preferably with version 6.8.0 which we tested) plugin (or FIJI which is a version of ImageJ with an already integrated Bio-Formats plugin).

The minimal requirements for both are Windows XP or later with Java installed, Mac OS X 10.8 or later with Java installed, Ubuntu Linux 12.04 LTS, or later with Java installed. Minimal RAM is based on the size of processed images. In this case, multiple images are opened at once.

## Use cases

The usability of the introduced tools is presented in the next sections. A use case for comparison for a CT measured with different slicing setup is presented. Results for a set of 5 CTs evaluated by different users is discussed. Since we were restricted by hardware, two versions of tool were created. One that works with 8-bit version of images and needs less RAM, and one that works with 16-bit signed images and can load HU units. The CT scans of COVID-19 patients used in this section were provided by the Department of Radiology of Faculty hospital Královské Vinohrady, where the tool was tested and deployed in September 2021.

### Slice thickness variation

The international standard for saving DICOM files defines 3 mm slicing of CT data as the default way. However resaving data as TIFF (losing voxel information) or using different slice thicknesses (like 0.6 mm slicing) may result in a different result. In theory, 0.6 slicing would provide 5 times more detailed sampling in the Z-axis. However, in practice it is different.

**Figure 3.**
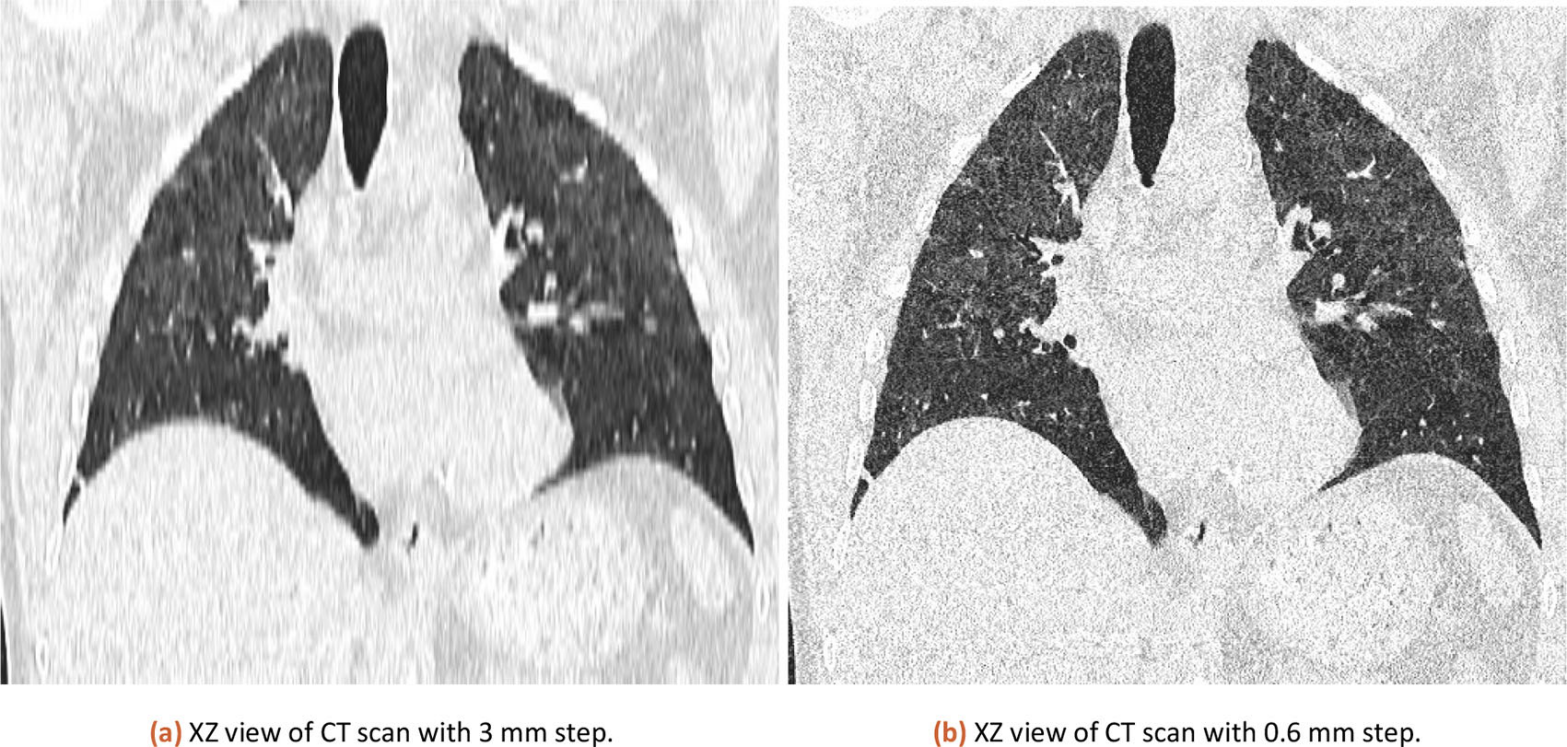
XZ view comparison of 3 mm and 0.6 mm CT.

The same CT dataset exported with 0.6 and 3 mm slices (XZ view for comparison is in Figure 3) was analyzed with our tool with a lung threshold of 0-155 and a pneumonia threshold of 47-115. The results can be found in Table 1. The error from a comparison of 3 mm and 0.6 mm slicing is estimated at 0.58 %. The used CT is available in the attached published dataset as CT1_1 (0.6 mm slicing) and CT1_2 (3 mm slicing).

**Table 1.** Comparison of results from 0.6 mm and 3 mm 8-bit dataset.

Slicing	Lungs slices	Lungs threshold	Pneumoina threshold	Percentage	Scoring
0.6 mm	60-505	0-155	47-115	31.21	3
3 mm	12-101	0-155	47-115	31.79	3

### User inter and intra variability

The biggest challenge in using this tool is an individual perception of images, as each person may see image data fundamentally the same - despite different appearances. Based on this a user can add the biggest bias even though the underlying data analysis is done correctly. The Table 2 contains a comparison of the results of the analysis in on 5 different CT datasets provided by the Faculty hospital of Královské Vinohrady. All CTs are analysed by users with different experience. The first CT exported with different slicing (also used in Table 1) is analysed by a radiologist (an expert user). The ANOVA test ( Table 5 Results for Score, Table 6 Results for Percentage) assesses the variance between datasets is statistically significant ( **For Score** F-value: **75.06** and p-value: **1.64e-21**, **For Percentage** F-value: **89.85** and p-value: **3.53e-23**), proving the selection of datasets is representative for comparisons.

The score aims to divide the percentage into groups based on previous research done, ^13^ and should be the deciding factor for future care for patients.

**Table 2.** Independent analysis results using the 8bit version of tool, example logs available at github.com/martinschatz-cz/ImageJ_Pneumonia_Estimation_Tool.

Dataset	Slicing	Radiologist	Rad. score	User 1	User 1 score	User 2	User 2 score	User 3	User 3 score
CT1_1	0.6 mm	31%	3	50%	3	30%	3	30%	3
CT1_2	3.0 mm	32%	3	55%	4	33%	3	45%	3
CT2	3.0 mm	-	-	10%	2	5%	1	7%	1
CT3	0.6 mm	-	-	41%	3	24%	2	42%	4
CT4	3.0 mm	-	-	64%	4	64%	4	64%	4
CT5	3.0 mm	-	-	2%	1	3%	1	4%	1

**Table 3.** Scoring results for 3 repetitions.

Run 1			Run 2			Run 3		
Dataset	Score	Percentage	Dataset	Score	Percentage	Dataset	Score	Percentage
CT1_1	3.0	32.11	CT3	3.0	30.00	CT1_1	2.0	23.12
CT1_1	3.0	42.14	CT2	2.0	20.72	CT1_1	3.0	50.68
CT2	2.0	18.15	CT5	1.0	1.06	CT1_2	4.0	59.63
CT4	4.0	74.76	CT1_1	3.0	32.84	CT2	1.0	4.62
CT3	3.0	37.70	CT3	3.0	42.95	CT3	3.0	43.24
CT1_1	3.0	32.83	CT1_1	3.0	45.15	CT4	4.0	69.70
CT2	2.0	11.79	CT4	4.0	72.60	CT3	3.0	30.62
CT5	1.0	2.84	CT1_2	3.0	36.31	CT2	2.0	9.56
CT4	4.0	71.12	CT5	1.0	1.23	CT4	4.0	69.18
CT3	3.0	28.11	CT1_1	3.0	48.35	CT1_2	3.0	52.55
CT4	4.0	64.64	CT2	2.0	15.45	CT2	1.0	5.14
CT3	3.0	34.76	CT4	4.0	71.02	CT4	4.0	70.17
CT5	1.0	2.21	CT2	2.0	19.45	CT5	1.0	0.14
CT2	2.0	19.59	CT1_2	2.0	25.45	CT1_1	3.0	47.73
CT5	1.0	1.62	CT3	3.0	35.46	CT5	1.0	0.48
CT1_2	3.0	49.25	CT1_2	2.0	22.72	CT3	3.0	33.72
CT1_2	3.0	42.38	CT5	1.0	2.94	CT5	1.0	0.52
CT1_2	3.0	51.98	CT4	4.0	65.34	CT1_2	4.0	55.97

**Table 4.** Standard Deviation and CV for Each Metric.

Filename	mean	std	cv	metric
CT1_1	2.888889	0.333333	0.115385	Score
CT1_2	3.000000	0.707107	0.235702	Score
CT2	1.777778	0.440959	0.248039	Score
CT3	3.000000	0.000000	0.000000	Score
CT4	4.000000	0.000000	0.000000	Score
CT5	1.000000	0.000000	0.000000	Score
CT1_1	39.438889	9.497811	0.240823	Percentage
CT1_2	44.026667	13.278828	0.301609	Percentage
CT2	13.830000	6.287444	0.454624	Percentage
CT3	35.173333	5.381494	0.152999	Percentage
CT4	69.836667	3.215369	0.046041	Percentage
CT5	1.448889	1.029629	0.710634	Percentage

**Figure 4.**
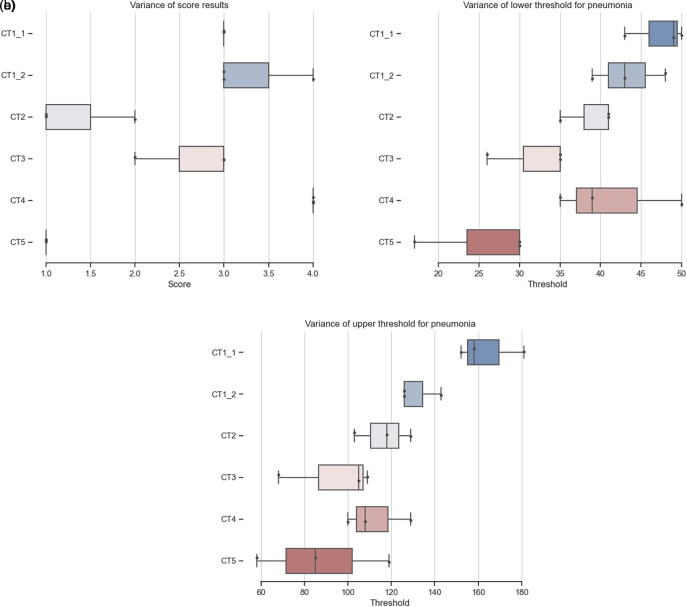
Comparison of CT scoring and threshold in between users. More details and code is available at https://github.com/martinschatz-cz/ImageJ_Pneumonia_Estimation_Tool. (a) Distribution of CT scoring from three users, Table 2. (b) Distribution of lower threshold over three users. (c) Distribution of upper threshold over three users.

Ensuring the reliability and accuracy of results when working with user-input tools is crucial and requires careful consideration of inter- and intra-variability. This challenge can be addressed by using standardized procedures and guidelines, multiple raters for segmentation, and computer-aided methods. Our software tool addresses this issue by providing standardized procedures and guidelines, along with the ability to compare results through logs and promote reproducibility.

It is essential to take into account the level of training and experience of individuals performing the segmentation, as well as the time and resources available, as these factors can significantly impact the consistency and accuracy of the segmentation. The software tool provides a solution for addressing the challenges of inter and intra variability in CT data segmentation, helping to ensure careful planning and execution of a study and appropriate outputs to achieve repeatable and comparable results.

Using scoring will overcome some of the problems of comparing percentages directly. Figure (Comparison of CT scoring) shows that users rely on their experience and will choose parameters based on them. It shows the difficulties in ensuring consistency and accuracy of data segmentation when performed manually by multiple individuals. The possibility of comparing the results of the analysis of multiple users using a defined analysis process ( Figure 4a) leads to more reliable results. CT1_1 and CT1_2 is the same dataset with different slicing and percentage results of analysis from all users clearly show inter-variability ( Table 2, thresholds and scores in Figure 4). The overall scoring is the same as the result from the trained radiologists (CT1_1 - 31%, score 3; CT1_2 - 32%, score 3).

Intra variability ^15^ was established by 3 runs of set of randomized and blinded datasets. Each run consisted of 3 copies of the 6 published datasets, in random order. There was a at least 4 weeks in between of evaluation of each run. The resulting Statistical Analysis consists of the standard deviation and coefficient of variation for each metric (percentage and score) across repetition and an ANOVA ( Table 5 Results for Score and Table 6 Results for Percentage) and intraclass correlation coefficient (ICC) analysis to assess agreement and consistency. The results can be found in Table 3 Scoring results for 3 repetitions.

*Tool performance based on descriptive stats*

The single-user scoring system demonstrates excellent reliability (ICC1 = 0.91), showing that the user's scoring using proposed toll is consistent across datasets. This reliability is critical since all dataset evaluations depend on the judgment of one rater using this tool. When we average scores across multiple evaluations (by the same user), the reliability improves even further (ICC1k = 0.97). This result indicates that averaging can mitigate occasional inconsistencies and ensure stable, reproducible scoring. The high ICC values confirm that the scoring process is robust and reliable, even when performed by a single evaluator. This ensures the validity of the results and confidence in the conclusions drawn from the data analysis. The full ICC results are in Table 7 Results for Score and Table 8 Results for Percentage.

The tool still demonstrates varying precision across datasets ( Table 4 Standard Deviation and CV for Each Metric). Lower cv values for Percentage in CT4_TIFF and consistent Score in CT3_TIFF (cv: 0.00) suggest high reliability for these cases. Showing that previously designed Scoring parameters are well based, and preferred for use, while percentage can be valuable but not as reliable information. Because of this, user training, and using provided guidance will lead to stable results.

**Table 5.** ANOVA Results for Score.

Source	ddof1	ddof2	F	p-unc	np2
Filename	5	48	75.062069	1.639097e-21	0.886608

**Table 6.** ANOVA Results for Percentage.

Source	ddof1	ddof2	F	p-unc	np2
Filename	5	48	89.852298	3.532717e-23	0.903471

**Table 7.** ICC Results for Score.

Type	Description	ICC	F	df1	df2	pval	CI95%
ICC1	Single raters absolute	0.911795	32.011765	5	12	0.000002	[0.71, 0.99]
ICC2	Single random raters	0.911366	27.484848	5	10	0.000015	[0.69, 0.99]
ICC3	Single fixed raters	0.898253	27.484848	5	10	0.000015	[0.65, 0.98]
ICC1k	Average raters absolute	0.968761	32.011765	5	12	0.000002	[0.88, 1.0]
ICC2k	Average random raters	0.968600	27.484848	5	10	0.000015	[0.87, 1.0]
ICC3k	Average fixed raters	0.963616	27.484848	5	10	0.000015	[0.85, 0.99]

**Table 8.** ICC Results for Percentage.

Type	Description	ICC	F	df1	df2	pval	CI95%
ICC1	Single raters absolute	0.927952	39.639008	5	12	4.708720e-07	[0.75, 0.99]
ICC2	Single random raters	0.927662	33.969098	5	10	5.806596e-06	[0.74, 0.99]
ICC3	Single fixed raters	0.916595	33.969098	5	10	5.806596e-06	[0.7, 0.99]
ICC1k	Average raters absolute	0.974772	39.639008	5	12	4.708720e-07	[0.9, 1.0]
ICC2k	Average random raters	0.974666	33.969098	5	10	5.806596e-06	[0.89, 1.0]
ICC3k	Average fixed raters	0.970561	33.969098	5	10	5.806596e-06	[0.88, 1.0]

## Discussion

The ImageJ/FIJI tool can import various DICOM or TIFF files. Users should be always aware of whenever the saved data are using signed or unsigned bit depth, as unsigned data will shift pixel brightness. The same will happen when exporting data in different bit depth or with a specific CT view. The slicing of the CT dataset also matters, however, the analysis in Table 1 showed that it won’t significantly affect either the percentage or the score (other CT machines might have different settings). A small case study for user inter and intra variability was made ( Table 2) to evaluate the usability of the proposed tool. Some expected variability in results occurs, interesting is inter variability in evaluating CT1 which is 3-5%. The intra variability is more extensive, up to 20%, and points out the fact that users should have at least some training in how to recognize pneumonia in CT images.

## Conclusions

The tool was developed on demand from the Department of Radiology at the Faculty hospital Královské Vinohrady, as it was difficult for them to estimate the percentage and score of pneumonia in the lungs just by visually inspecting CT scans. Available hardware equipment and local account restrictions had to be taken into account for development tool selection. The ImageJ plugin is a compromise in accuracy and requirements. It logs all the user inputs for reproducibility and saves the results of all the steps as TIFF stacks. These masks and images can be used for visual inspection or possibly in the future for more advanced machine learning tools.

This software tool is the first step of a longer journey to create a tool that would be both easy to use for radiologists to diagnose COVID-19 based on CTs and include an advanced image analysis tool for percentage estimation of pneumonia in lungs. The use of open software promises ease of future development, however, it might be beneficial to move from ImageJ to 3D Slicer ^12^ or Napari ^16^ as they offer better tools for 3D visualization and integration of machine learning tools, which we aim to develop and integrate into our future works.

### Limitations

The biggest limitation of this approach is human error and inter and intra variation of manual selection. The percentage estimation might also be affected by other body cavities filled with air. There might also be a variance in results based on slice thickness, in worst case scenario 20%, but our experiment shows that there is only about 0.58% difference in result between 0.6 and 3 mm CT slice thickness. The scoring should also be improved so it is not dependent only on one value (volume percentage), but normalized SHU distribution in the pneumonia area should be also considered. When converting from 12-bit to 8-bit image representation, the reduced range of values results in a loss of information and detail, which can lower the quality of the output. However, for CT image segmentation, the use of Single Hounsfield Unit (SHU) values is adequate, as SHUs do not rely on single units and can provide good-quality segmentation.

For evaluating the agreement between repeated measurements, we used the **intraclass correlation coefficient (ICC)**. ICC is a widely used indicator of reliability in medical research. ^15^ However, it is known that **the ICC value is sensitive to the range of variability in the measured sample**. ^15^ A narrow range of true values in the sample can lead to a low ICC even with good measurement agreement, and conversely, a wide range can artificially inflate the ICC value. Therefore, when interpreting our ICC results, it is necessary to consider the **potential influence of data range**. Our decision to use ICC was motivated by its **common use for evaluating inter- and intra-rater reliability** and the desire for comparability with existing literature in the field of image data analysis.

From the software point of view, there is a limitation in the version of ImageJ used. The new version of the code logs the ImageJ version and BioImage plugin version. There is a version of the code explicitly made for ImageJ version 1.52v99 and for other versions. The bind version helps reproducibility of any analysis based on logs, and it is advised to reproduce the analysis in the same version of ImageJ as indicated in logs.

## Data availability

### Underlying data

Zenodo: CT scans of COVID-19 patients, https://doi.org/10.5281/zenodo.5805939. ^17^

Datasets contain CT scans of COVID-19 patients from Faculty hospital of Královké Vinohrady in DICOM (and TIFF), as per the folder name. Dataset CT1 is presented with 0.6 mm and 3 mm slicing.

This project contains the following underlying data:

• CT1_1
  – CT1_1_TIFF_06_MM (Single stack 8-bit TIFF data)
• CT1_2
  – CT1_2_TIFF_3_MM (Single stack 8-bit TIFF data)
• CT2
  – CT2_DICOM
  – CT2_TIFF (Single stack 8-bit TIFF data)
• CT3
  – CT3_DICOM
  – CT3_TIFF (Single stack 8-bit TIFF data)
• CT4
  – CT4_DICOM
  – CT4_TIFF (Single stack 8-bit TIFF data)
• CT5
  – CT5_DICOM
  – CT5_TIFF (Single stack 8-bit TIFF data)
• results_csv.csv

Data are available under the terms of the Creative Commons Attribution 4.0 International (CC-BY 4.0).

## Software availability

Zenodo: ImageJ tool for percentage estimation of pneumonia in lungs, https://doi.org/10.5281/zenodo.15827771. ^18^

The third version of the repository contains both a new version of ImageJ scripts (.ijm files in folder **tools**) and ImageJ scripts published with the first version of this Software Tools article (subfolder **0.3c1** of folder tools). The new folders **inter**_intra and **repeatability** contains the source files and Jupyter Notebook files used for the evaluation and the presented graphs.

The repository is accompanied by overview of project, limitations and step-by-step tutorial using the CT3 dataset published as a part of the underlying data. All this information is also available on GitHub repository: https://github.com/martinschatz-cz/ImageJ_Pneumonia_Estimation_Tool. This supplementary material aims to improve reproducibility and with the community approved way to publish workflows. ^19^

This project structure describes the crucial underlying data:

• Inter_intra
○ **Inter_variability**
▪ user_eval.ipynb
▪ users_result.csv
○ **Intra_variability**
▪ intra_var_analysis.ipynb
▪ Results_1.csv
▪ Results_2.csv
▪ Results_3.csv
• repeatability
○ score_eval
○ time_eval
• tools
○ SEQUENCE_Est_Percentage_CT_16bit_V04_IJ_152v99.ijm
○ SEQUENCE_Est_Percentage_CT_u8bit_V04_IJ_152v99.ijm
○ SEQUENCE_Est_Percentage_CT_u8bit_V04.ijm
○ 0.3c1
▪ SEQUENCE_Est_Percentage_CT_16bit_V03_clean.ijm (16bit version)
▪ SEQUENCE_Est_Percentage_CT_u8bit_V03_clean.ijm (8bit version)
• README.md

Data are available under the terms of the Creative Commons Attribution 4.0 International license (CC-BY 4.0).
