# Supplementary material for: Estimation of Covid-19 lungs damage based on computer tomography images analysis
Source: F1000Res. 2025 Jul 25;11:326. Originally published 2022 Mar 17. [Version 3] doi: 10.12688/f1000research.109020.3 (PMC12365592; doi:10.12688/f1000research.109020.3)
Supplement: Peer review history

# Reviewer report: Reviewer response for version 3

**DOI:** 10.5256/f1000research.184052.r400406

**Published:** 2025-08-19

**Authors:** Alessandro Santini

**Affiliations:** 1 Department of Biomedical Sciences, Humanitas University, Milan, Italy

I thank the Authors for their review and update of the manuscript, which I feel is now suitable for indexing.

Are the conclusions about the tool and its performance adequately supported by the findings presented in the article?

Partly

Is the rationale for developing the new software tool clearly explained?

Yes

Is the description of the software tool technically sound?

Yes

Are sufficient details of the code, methods and analysis (if applicable) provided to allow replication of the software development and its use by others?

Yes

Is sufficient information provided to allow interpretation of the expected output datasets and any results generated using the tool?

Partly

Reviewer Expertise:

Acute respiratory distress syndrome, ventilator-induced lung injury, critical care medicine

I confirm that I have read this submission and believe that I have an appropriate level of expertise to confirm that it is of an acceptable scientific standard.

# Reviewer report: Reviewer response for version 2

**DOI:** 10.5256/f1000research.147768.r211856

**Published:** 2023-11-01

**Authors:** Alessandro Santini

**Affiliations:** 1 Department of Biomedical Sciences, Humanitas University, Milan, Italy

The Authors present a new tool for quantitative analysis of CT scans of COVID-19 patients. The novelty of this tool, compared to already available softwares such as 3D Slicer, is its availability (the code is open-source) and the alleged ease of use even by non-experienced users.

 However, when presenting results of inter- and intra-user variability of results, the Authors show not very promising results. They also acknowledge this problem in the discussion: "The biggest challenge in using this tool is an individual perception of images (...) Based on this a user can add the biggest bias even though the underlying data analysis is done correctly". While I agree with the Authors, I do not see how this problem is overcome by their tool. The Authors later in the manuscript state that "this challenge can be addressed by using standardized procedures and guidelines, multiple raters for segmentation, and computer-aided methods". However, it is not clear from this paper that this challenge has efficiently being addressed/solved.

 This is not a secondary issue for a tool which is designed to be "user-friendly" and easy to use even by a non-experienced user.

 I suggest the Authors to expand their inter- and intra-user variability analysis. While inter-user variability is presented, I did not find any data on intra-user variability (same user performing the analysis on a single CT more than once). You can use as a guide the following ref: Popović ZB and Thomas JD "Assessing observer variability: a user’s guide" Cardiovasc Diagn Ther. 2017 Jun; 7(3): 317–324.

 Furthermore, as the tool is supposed to give the same results as other, already available, softwares, the Authors should present a comparison between the results obtained with their tool and the results obtained with other(s) software(s) on their CT scans.

Are the conclusions about the tool and its performance adequately supported by the findings presented in the article?

Partly

Is the rationale for developing the new software tool clearly explained?

Yes

Is the description of the software tool technically sound?

Yes

Are sufficient details of the code, methods and analysis (if applicable) provided to allow replication of the software development and its use by others?

Yes

Is sufficient information provided to allow interpretation of the expected output datasets and any results generated using the tool?

Partly

Reviewer Expertise:

Acute respiratory distress syndrome, ventilator-induced lung injury, critical care medicine

I confirm that I have read this submission and believe that I have an appropriate level of expertise to confirm that it is of an acceptable scientific standard, however I have significant reservations, as outlined above.

## References

[1] : Assessing observer variability: a user's guide. *Cardiovasc Diagn Ther* .2017;7(3) : 10.21037/cdt.2017.03.12 317-324 10.21037/cdt.2017.03.12 28567357 PMC5440257

# Response

**Published:** 2023-11-21

**Authors:** Martin Schätz

**Affiliations:** University of Chemistry and Technology, Prague, Czech Republic

Dear Dr. Alessandro Santini,

 we express our sincere gratitude for your diligent review of our manuscript and for providing constructive and insightful comments.

 We wish to address your suggestion for the comparison of our tool with other software tools first. Numerous freeware programs exist for visualizing scientific image data, such as Imaris Viewer ( https://imaris.oxinst.com/imaris-viewer), ZEISS ZEN lite ( https://www.zeiss.com/microscopy/en/products/software/zeiss-zen-lite.html), and open-source software like 3D Slicer and FIJI/ImageJ, which offer manual BioImage Analysis and script creation capabilities. Analogous to CT software, these applications typically afford scientific data visualization, with open-source variants featuring additional tools or plugins for specific and systematic analyses. While we appreciate the suggestion to compare our tool with the Slicer Lung CT Analyzer extension of 3D Slicer, it is important to note that, as of the current date (21.11.2023), the extension is still in development and remains unpublished (you can find details on their GitHub repository: https://github.com/rbumm/SlicerLungCTAnalyzer). We eagerly anticipate the extension's publication and look forward to a thorough comparison, as their design is sound, and offers a good alternative in case of access to the proper hardware. Publishing software tools takes time and when Slicer Lung CT Analyzer extension is published, it deserves a proper citation and acknowledgment.

 We value your feedback regarding the improvement of the use case and the suggestion to expand the paper more. To clarify our aim for user-friendliness, the tool was developed with the goal in mind, that „guessing“ volumetric percentage out of CT viewer is not an exact quantitative analysis. While manual analysis in other software is possible after user training, our tool's systematic approach, documented parameters, and step-by-step guide enable hospital staff to apply it with reasonable variability. During the tool's pilot implementation at FNKH Faculty Hospital, our focus was on lowering inter-user variability, as reflected in our report. However, what was tested was a score comparison between CT of the same patient with different slice thicknesses (CT1_1 with 0.6mm slices and CT1_2 with 3 mm slices). It was originally designed to assess score variability with different slice thicknesses. This comparison also provides insight into intra-user variability; however, we acknowledge that the number of repetitions may have some limitations.

 Once again, we sincerely appreciate your invaluable feedback.

 Best regards,

 Martin Schätz

# Response

**Published:** 2025-07-07

**Authors:** Martin Schätz

**Affiliations:** University of Chemistry and Technology, Prague, Czech Republic

Dear reviewer,

 thank you very much for insightfull feedback and recomendations to improve our analysis of our tool.

**Addressing User Bias and Tool's Focus**

 We agree that user perception introduces inherent variability in manual segmentation, a challenge common to all semi-automatic tools. Our tool addresses this not by eliminating user bias entirely, but by simplifying the segmentation process, making it more accessible and reducing errors from completely manual focus often used in hospitals. Its primary focus is on providing a standardized framework for quantitative analysis. This approach, coupled with full reproducibility through detailed logging of all sub-results, is particularly beneficial in a hospital setting where multiple raters often compare results. This was our key motivation for initially focusing on inter-user variability, as a single rater typically doesn't repeat the analysis on the same scan in clinical practice.

**Intra- and Inter-User Variability Analysis**

 We appreciate the suggestion to expand our variability analysis. We've now included both intra-user and inter-user variability analyses in the updated manuscript. Jupyter notebooks with analysis, data and subresults are included in Zenodo and also available on GitHub, which is more user friendly for browsing such files. We would like to thank you for the helpful reference (Popović ZB and Thomas JD "Assessing observer variability: a user’s guide" Cardiovasc Diagn Ther. 2017 Jun; 7(3): 317–324), which guided our approach and is cited in the paper.

**Comparison with Existing Software**

 Our tool is specifically developed for quantitative analysis in a hospital environment. This setting is often **strict about software installation** and may **lack the necessary hardware to run complex neural network or deep learning models efficiently**, if at all. While we acknowledge the importance of comparing our results with other published software, the specific 3D Slicer plugin relevant to our approach is still under development, as noted in its GitHub repository ( https://github.com/Slicer/SlicerLungCTAnalyzer). We plan to conduct a thorough comparison with this and potentially other tools as they become finalized and published. At the moment the creators of this tool recomends/references general CT neural network segmenters which are useful for lung segmentation or segmentation of anatomic structures from CT. Unfortunately, that is only substep in whole analysis workflow.

 We agree the comparison is a crucial point for future versions and further analysis of our tool.

# Reviewer report: Reviewer response for version 2

**DOI:** 10.5256/f1000research.147768.r183705

**Published:** 2023-07-18

**Authors:** Tamas Dolinay

**Affiliations:** 1 Department of Medicine, University of California, Los Angeles, Los Angeles, CA, USA

The authors have sufficiently revised the manuscript. I believe the revised paper is an important addition to the growing field of CT imaging in COVID-19 lung disease.

Are the conclusions about the tool and its performance adequately supported by the findings presented in the article?

Yes

Is the rationale for developing the new software tool clearly explained?

Yes

Is the description of the software tool technically sound?

Yes

Are sufficient details of the code, methods and analysis (if applicable) provided to allow replication of the software development and its use by others?

Yes

Is sufficient information provided to allow interpretation of the expected output datasets and any results generated using the tool?

Yes

Reviewer Expertise:

Pulmonology

I confirm that I have read this submission and believe that I have an appropriate level of expertise to confirm that it is of an acceptable scientific standard.

# Reviewer report: Reviewer response for version 1

**DOI:** 10.5256/f1000research.120473.r153263

**Published:** 2022-10-25

**Authors:** Hamid A. Jalab

**Affiliations:** 1 hamidjalab@um.edu.my Hamid A. Jalab Department of Computer System and Technology, Faculty of Computer Science and Information Technology, Universiti Malaya, Kuala Lumpur, Malaysia

The study describes a novel image tool developed for CT assessment of COVID-19 patients. The tool was created to assist in estimating the percentage of lungs infected by the virus.

 The manuscript is interesting and addresses an important issue. Major comments:

 1. The Introduction should provide a strong argument for why the software tool is important.

 2. It is of importance to have sufficient results to justify the novelty of the proposed software tool.

 3. The robustness of the proposed software tool has not been addressed; this should be emphasized in the discussion section.

 4. What are the benefits of the proposed approach above other current the new software tool?

Are the conclusions about the tool and its performance adequately supported by the findings presented in the article?

No

Is the rationale for developing the new software tool clearly explained?

No

Is the description of the software tool technically sound?

No

Are sufficient details of the code, methods and analysis (if applicable) provided to allow replication of the software development and its use by others?

No

Is sufficient information provided to allow interpretation of the expected output datasets and any results generated using the tool?

No

Reviewer Expertise:

image processing

I confirm that I have read this submission and believe that I have an appropriate level of expertise to state that I do not consider it to be of an acceptable scientific standard, for reasons outlined above.

# Response

**Published:** 2023-05-05

**Authors:** Martin Schätz

**Affiliations:** University of Chemistry and Technology, Prague, Czech Republic

Dear Dr. Hamid A. Jalab, 

 I am extremely grateful for the time and effort you have dedicated to reviewing our Software Tool Article. Your constructive comments have been instrumental in shaping our manuscript, and I truly appreciate your contributions to the peer review process.

 Our replies to your comments: 

**1. The Introduction should provide a strong argument for why the software tool is important.**

 Thank you for your valuable feedback. We agree that the Introduction should provide a strong argument for the software tool's importance. As we highlighted in the new paragraph, there is a need for a open source, user-friendly software tool for reproducible quantitative analysis of CT scans to estimate COVID lung pneumonia. Current software tools such as ImageJ and 3D Slicer may not be user-friendly for end-users who are not familiar with creating analysis workflows. Our software tool aims to fill this gap by providing a user-friendly solution for reproducible quantitative analysis, with available code, training data, tutorial and GitHub repository. 

**2. It is of importance to have sufficient results to justify the novelty of the proposed software tool.**

 The main motivation for the software tool was that no other similar software tool was available for use on the computer infrastructure of Faculty Hospital of Královské Vinohrady. The main obstacles were in internal network policy, the unavailability of any high performance computing hardware, and need of specific tool. Since more hospitals might be challenged in similar ways, the highest motivation was to share our work openly and freely to help innovate and enable them. 

**3. The robustness of the proposed software tool has not been addressed; this should be emphasized in the discussion section.**

 Since the software tool is user operated, there is high inter and intra variability of results – which we addressed in text. The robustness of whole workflow is now addressed in terms of repeatability. 

**4. What are the benefits of the proposed approach above other current the new software tool?**

 The only other open-source software tool adressing similar medical analysis is plugin SlicerLungCTAnalyzer for 3D Slicer software (available on GitHub: https://github.com/rbumm/SlicerLungCTAnalyzer). Of course there also exists commercially available software like Thoracic VCAR and others, but the money and hardware requirements might render these software unreachable for a lot of facilities, and since the tools are closed it might be enough for diagnosis but not for other research.

 The benefits of our Software tool are that it is developed open-source, in its very low hardware requirements and its ease of use with low technical requirements for installation. These were also the requirements of the faculty hospital where it was requested. 

- **Is the rationale for developing the new software tool clearly explained? **

We added a comparison to other freely available open source software tool as requested. 

- **Is the description of the software tool technically sound? **

We improved the pseudo-code on top of the available source code, which should improve the description overview of the whole tool. 

- **Are sufficient details of the code, methods and analysis (if applicable) provided to allow replication of the software development and its use by others? **

The whole original code and its update is available through a linked (now updated) repository and on GitHub with a small tutorial. The data and software availability parts were extended. We added pseudocode for a better overview, but best option is to go through the code. Any encountered problems with the macro on specific machines and/or ImageJ version can be reported through an issue on GitHub repository. 

- **Is sufficient information provided to allow interpretation of the expected output datasets and any results generated using the tool? **

The output of the software tool is a log of whole process and partial results, it is a necessary step for reproducibility, good data management and interpretation. 

 If you encountered any issues with the tool or prepared protocols please report them in GitHub repositories so we can properly address them. We are happy to help, sustainability is important part of our project. 

- **Are the conclusions about the tool and its performance adequately supported by the findings presented in the article? **

We included more analysis about performance of the tool and also inter and intra variability overview (also available on GitHub). Scripts are included in linked materials with both new version of Software Tool. 

 In the meantime we did optimization and some correction of reported issues of our ImageJ scripts, you are able to find them in the linked GitHub repository. It involves time and result-robustness tests , inter and intra variance overview from reported parameters used, a new version including updated log protocols to help users troubleshoot possible issues with the version of ImageJ. New logs from both 8 and 16-bit versions of scripts now contain both the ImageJ version used and Bio-Image version used. We will welcome any issue reports or enhancement suggestions in the GitHub repository. 

 Thank you again for your valuable feedback. 

 Martin Schätz

# Reviewer report: Reviewer response for version 1

**DOI:** 10.5256/f1000research.120473.r146294

**Published:** 2022-08-10

**Authors:** Tamas Dolinay

**Affiliations:** 1 Department of Medicine, University of California, Los Angeles, Los Angeles, CA, USA

Dr. Schätz and colleagues describe a new image analysis modality to quickly review and score lung CT scan images. The image analysis was applied to patients with COVID-19 pneumonia to aid with diagnostic accuracy. It is increasingly recognized that viral pneumonias, including COVID-19-associated pneumonia, may have a specific radiographic pattern. Automated modalities, including the one described by the authors can help  with the rapid identification viral pneumonias and  with measurement of the extent of the disease.

 The manuscript is interesting and covers an important topic, but its current format suffers from a few weaknesses.

 Major comments:

1. The abstract describes 5 different pattern of radiographic findings, but I do not find these in the manuscript. A novelty of the article would be the stratification of pneumonias based on the imaging.
2. Please describe, if you had success implementing the image analysis in your clinical practice. What are the benefits and/or barriers of using the analysis in the real world?

Minor comments: Please write out the abbreviation when used for the first time to help the reader, who is not necessarily familiar with the specific language used.

Are the conclusions about the tool and its performance adequately supported by the findings presented in the article?

Yes

Is the rationale for developing the new software tool clearly explained?

Yes

Is the description of the software tool technically sound?

Yes

Are sufficient details of the code, methods and analysis (if applicable) provided to allow replication of the software development and its use by others?

Yes

Is sufficient information provided to allow interpretation of the expected output datasets and any results generated using the tool?

Yes

Reviewer Expertise:

Pulmonology

I confirm that I have read this submission and believe that I have an appropriate level of expertise to confirm that it is of an acceptable scientific standard, however I have significant reservations, as outlined above.

# Response

**Published:** 2023-05-05

**Authors:** Martin Schätz

**Affiliations:** University of Chemistry and Technology, Prague, Czech Republic

Dear Dr. Tamas Dolinay, 

 I want to express my sincere gratitude for taking the time to review our work and providing us with such thoughtful feedback. Your comments have helped us to strengthen our work significantly, and I appreciate your efforts.

 Our replies to your major comments: 

**1) The abstract describes 5 different pattern of radiographic findings, but I do not find these in the manuscript. A novelty of the article would be the stratification of pneumonia based on the imaging.**

 We appreciate your comments and would like to address your concern about the radiographic findings described in the abstract. The 5 groups are distinguished based on the percentage coverage in the lungs. We appologize for the confusion. We will revise the abstract to more accurately reflect your comment. 

 The 5 different scores are originally based on paper „Chest CT score in COVID-19 patients: correlation with disease severity and short-term prognosis“ (https://doi.org/10.1007/s00330-020-07033-y) where the lungs are divided and the result of each part is summed up to the score. It would be possible to do the same with 3D Slicer and the „Lung CT Analyzer“ (https://github.com/rbumm/SlicerLungCTAnalyzer) deep learning tool, however, it would require installing it and having more powerful hardware available. The software tool paper aims to present software tool that was made per request of Faculty Hospital, however analysis of different pattern of radiographic findings would be quite an interesting suggestion for a full research article. 

**2) Please describe, if you had success implementing the image analysis in your clinical practice. What are the benefits and/or barriers of using the analysis in the real world?**

 The ImageJ software tool was developed on request by the Facultypital of Královské Vinohrady, and it has been used by the Radiology center since September 2021. The score was mainly used for additional estimation to help doctors estimate the severity of COVID-19 disease in hospitalized patients. The first benefit is having a quick estimation of lung coverage by disease, which can be challenging to estimate by visual inspection. The second benefit is that the ImageJ and this specific SW Tool do not need installation (which can be challenging at the hospital IT infrastructure) and works on a average office computer. The challenge is mentioned inter and intra variability per user, but logging the whole process helps with that. 

 In meantime we did optimization and some correction of reported issues of our ImageJ scripts, you are able to find them in the linked GitHub repository. It involves time and result robustness tests , inter and intra variance overview from reported parameters used, a new version including updated log protocols to help users troubleshoot possible issues with the version of ImageJ. New logs from both 8 and 16-bit versions of scripts now contain both the ImageJ version used and Bio-Image version used. We will welcome any issue reports or enhancement suggestions in the GitHub repository. 

 Thank you again for your valuable feedback. 

 Martin Schätz
